# Structural, Chemical and Magnetic Characterization of Quartz Sand from Cluj Area, Romania for Future Beneficiation in the Glass Industry

**DOI:** 10.3390/ma15249026

**Published:** 2022-12-17

**Authors:** Florin Popa, Traian Florin Marinca, Bogdan Viorel Neamțu, Mihai Gabor, Ionel Chicinaş

**Affiliations:** 1 Department of Materials Science and Engineering, Technical University of Cluj-Napoca, 103–105 Muncii Avenue, 400641 Cluj-Napoca, Romania; 2 Center for Superconductivity, Spintronics and Surface Science, Physics and Chemistry Department, Technical University of Cluj-Napoca, Memorandumului No. 28, 400114 Cluj-Napoca, Romania

**Keywords:** quartz sand, iron impurity, electron microscopy, magnetism

## Abstract

For use in crystal glass production, quartz sand must contain less than 0.09% iron. If the sand contains more than 0.09% Fe, the iron must be removed. In the present study, quartz sand from tailings ponds near the Cluj area of Romania is analyzed for potential use in the glass industry, after magnetic separation. The particle size distribution of raw sand was determined, and mineralogical analyses was realized. Using scanning electron microscopy (SEM) and energy dispersive X-ray spectroscopy (EDX), morphology and elemental distribution maps on the particle was performed. The evolution of the iron content versus the particle size was analyzed. Using X-ray diffraction, the phases occurring in the sand were investigated in relation to the particle size. Magnetic separation with two magnets, having different energy, was performed for identifying the phases attached to the magnetic particles. Magnetic hysteresis measurements evidenced complex and multiple iron phase behavior.

## 1. Introduction

High purity quartz finds its demand in a wide range of domains: glass, lighting, semiconductor, photovoltaic and optical fibers [1]. However, not all quartz sands are suitable for use in the glass industry. The major impediment is the iron content. If a quantity of more than 0.1% of iron is present, the resulting glass will be colored. The most used method for iron removal is flotation. For an efficient recovery, the proper reagents must be used, including NaOH, amine, or H_3_PO_4_ [2,3,4]. In the light of resource limitation and from environmental issues, a proper investigation of the available quartz sand resource is required. In addition, the potential of recycling different waste for use in glass industry was demonstrated [5].

Generally, iron appears in several forms in quartz sand resources: hematite (iron (III) oxide: Fe_2_O_3_); magnetite (naturally occurring iron oxides: Fe_3_O_4_); ilmenite (weakly magnetic titanium-iron oxide: FeTiO_3_), chromite (iron chromium oxide: FeCr_2_O_4_) and biotite (phyllosilicate mineral: K(Mg,Fe)_3_AlSi_3_O_10_(F,OH)_2_). Each type of iron compound contributes a certain susceptibility to the material mixture; higher susceptibility will give a higher magnetic response. The magnetic response of the material depends also on this concentration and if iron is represented by a larger mechanically blocked particle, or is an impurity phase in the sand grain [6].

For eliminating the undesired contamination, a first approach is to sieve the quartz sand and to perform optical sorting [1]. The importance of the particle size in application was evidenced in reference [7]. An easy way to obtain sand purification is presented in [8], whereby sieving the impurities were reduced and concentrated in low dimension particles. Higher iron contend (Fe_2_O_3_) is found to increase as the particle size decreases [9]. This result suggests the first way of sand purification: sieving [10]. However, the size reduction is not sufficient and supplementary processing is necessary. Processing ways imply either heat treatments [11] or grinding [12].

The understanding of sand reserves can be further advanced by performing X-ray diffraction (XRD) analysis. XRD offers an easy way for mineral identification, allowing to establish the phase purity [13,14,15]. Using X-ray diffraction and the Rietveld method, phase quantitative analysis can also be performed [16].

To complete the information about the phases contained, it is necessary to have an insight about the iron or element localization of the sand grains [17]. Such information about the phase and composition distribution, especially in low quantity percentage, can be obtained by performing Scanning Electron Microscopy (SEM) studies, coupled with energy dispersive X-ray spectroscopy, EDX. The importance of SEM in sand reserve characterization was evidenced in several studies [18,19,20,21,22]. The technique was also found beneficial for studying sand after mechanical reclaim [23].

Nowadays, sand is a natural reserve that can be processed to obtain high purity quartz sand by sieving, followed by magnetic separation. However, the amount of unused sand is important, but is not always a constraint. However, as transport and deposit costs increased, reserve particle size and mineralogical analysis became important. In this light, the present study wants to characterize the quartz reserves near the Cluj-Napoca aream in Romania. Most of these reserves represent tailings from other mining activities. The paper proposes an easy way to characterize the sand by X-ray crystallography, coupled with SEM/EDX analysis. In this way, chemical content as a function of grain size will be achieved. Information about the iron phase bonding (chemical or mechanical) will be considered for magnetic separation.

The magnetic properties were measured to study the particle size behavior in the magnetic field. Using the magnetic field response, two magnetic separation experiments were performed, using high and low magnetic fields. The resulting components were analyzed by X-ray diffraction.

## 2. Materials and Methods

The particle size analysis was performed on standard sieving method using 10 sieves in the range 40–800 µm, using the standard procedure. The standard sieved sand quantity was 100 g, loaded in a vibrating sieving apparatus for 30 min. Multiple dry sieving were performed to obtain statistical data. The resulting particle size ranges were morphological and compositional characterized by Scanning Electron Microscopy using a JEOL JSM 5600LV microscope (Tokyo, Japan) equipped with an EDX spectrometer (Oxford Instruments, INCA 200 software, AZtech 4.2 software (High Wycombe, UK). The SEM images were recorded in backscattered electron signal, under high vacuum at 15 kV accelerating voltage without coating the samples. Crystallographic analysis was performed by X-ray diffraction on a INEL 3000 Equinox diffractometer (Artenay, France), operating with CoKα radiation (λ = 1.79026 Å) in the angular range 2 theta of 20–110°. The magnetic hysteresis loops were recorded at room temperature on a Lake Shore Cryotronics Inc. vibrating sample magnetometer (VSM) (Westerville, OH, USA). The hysteresis loops were recorded with a 0.25 mT step and the maximum magnetic field was 18 kOe.

## 3. Results

**Figure 1:**
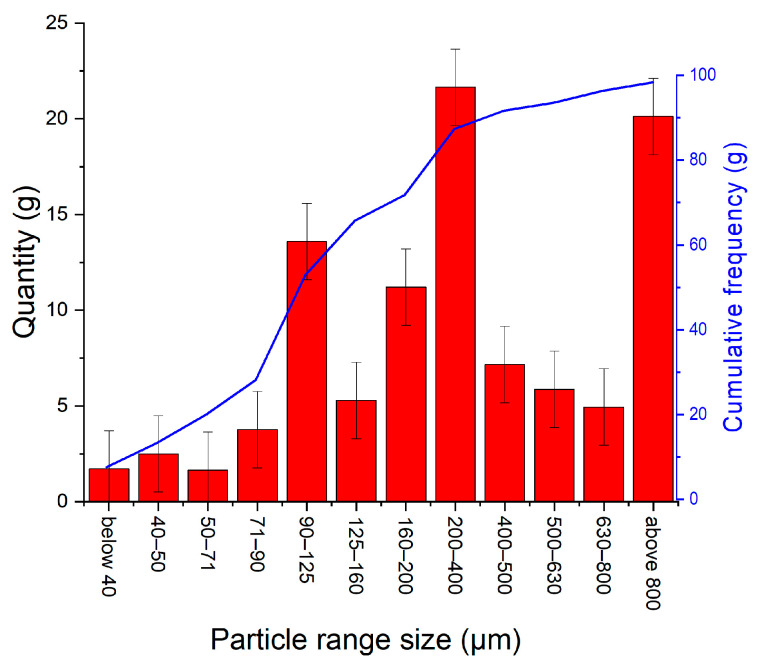
Particle size distribution of the quartz sand.

In the Cluj area, many quartz sand reserves can be found, either native or resulting from previous mining activities. For purification by magnetic separation, an understanding of the particle size distribution is necessary. The magnetic field necessary for iron phases removal is tailored by the particle size and the magnetic phase volume ratio in particles: if the iron phase has the same volume, the magnetic field strength necessary for magnetic phase removal will be higher for larger particle than for smaller ones. Figure 1 presents the particle size distribution of the target quartz sand. The particle size distribution indicates three major size ranges. About 20% from the total particle sizes presents dimensions larger than 800 μm, not considered for the present study, since additional processing is necessary—grounding, for example. A large quantity (21%) of particles sand has dimensions in the range 200–400 µm, and 23% of particles have dimensions smaller than 125 µm.

The second step in sand analysis is to connect the mineralogical analysis with particle size analysis. Table 1 summarizes the average quantitative results of the chemical analysis, performed on each particle size range.

**Figure 2:**
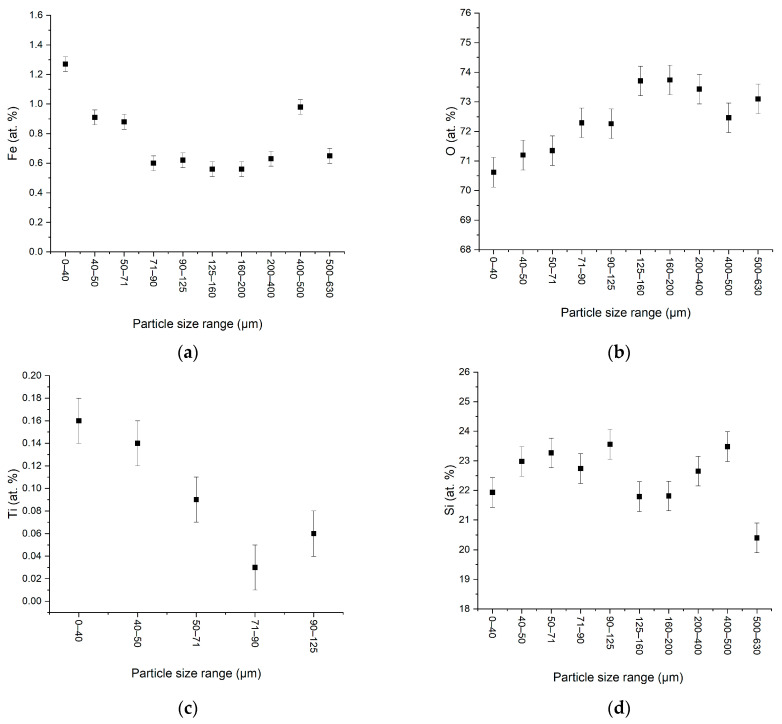
Elemental content of different particle size determined from the EDX analysis for Fe (**a**), O (**b**), Ti (**c**), and Si (**d**).

For the important elements (iron, oxygen, silicon and titanium) the obtained values versus the particle size are presented in Figure 2a–d.

The iron atomic percent presents a small variation for large particle sizes range (125–630 µm). In the small particle size the iron content increases, suggesting the iron compound as being small and connected with small silica particles. The iron compound increases in the low size range is accompanied by the oxygen decreases in this area. The oxygen decreases can be connected with the relative reduction of the quartz quantity and the preponderance of different other compounds. Indeed, the presence of other oxides with lower amount of oxygen as compared to quartz (Si to O ratio of 1:2) is suggested by this analysis. Many impurities, other phases present in the sand, described above, have the ratio among the metallic and non-metallic atoms to oxygen atoms lower (2:3 or 3:4). One possibility is the occurrence of FeSi compound suggested by the small variation of the Si in all particle size range. Supplementary, for the small particle size range, Ti is detected. Titanium can be present in sand either as titanium oxides or as ilmenite (FeTiO_3_).

**Figure 3:**
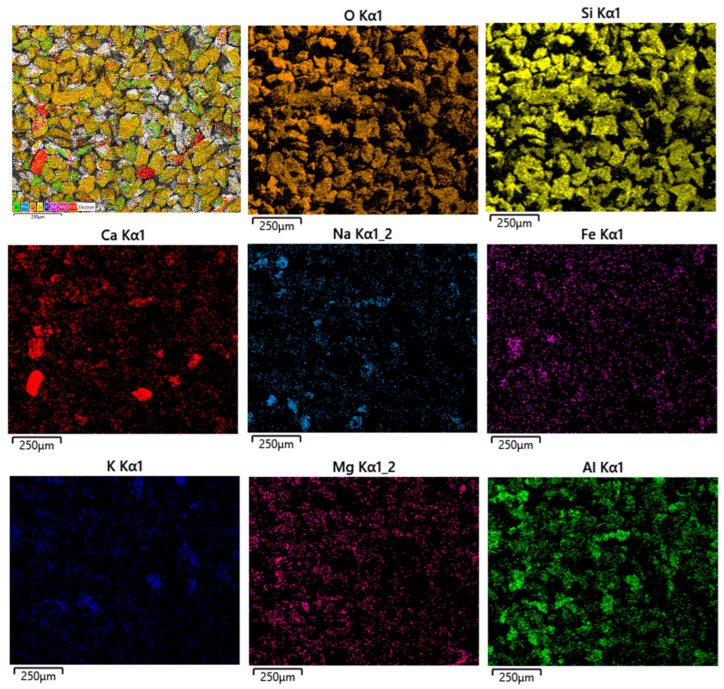
Elemental distribution maps for the quartz particle size ranging from 40 to 50 µm.

Having these compositions in mind, the distribution of the elements in the particles becomes important. For the 40–50 µm particle size range, the chemical map distribution of the elements is presented in Figure 3.

**Figure 4:**
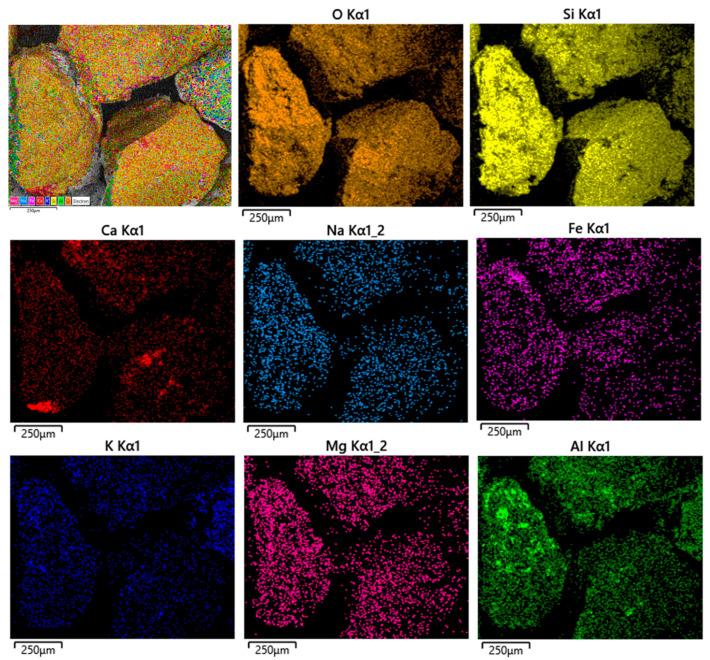
Elemental distribution maps for the quartz particle size ranging between 500 and 630 µm.

Chemical distribution maps for the 40–50 µm particle size range confirms the existence of several particles with different composition overall. The particles are mainly formed from K, Na and Ca. The occurrence of particles formed largely from light elements is also recorded for higher particle sizes up to 125 µm. For particles larger than 125 µm, the elements are mixed in the particles and form agglomerated structures. In Figure 4, the distribution of elements for samples in the range 500–630 µm is presented.

**Figure 5:**
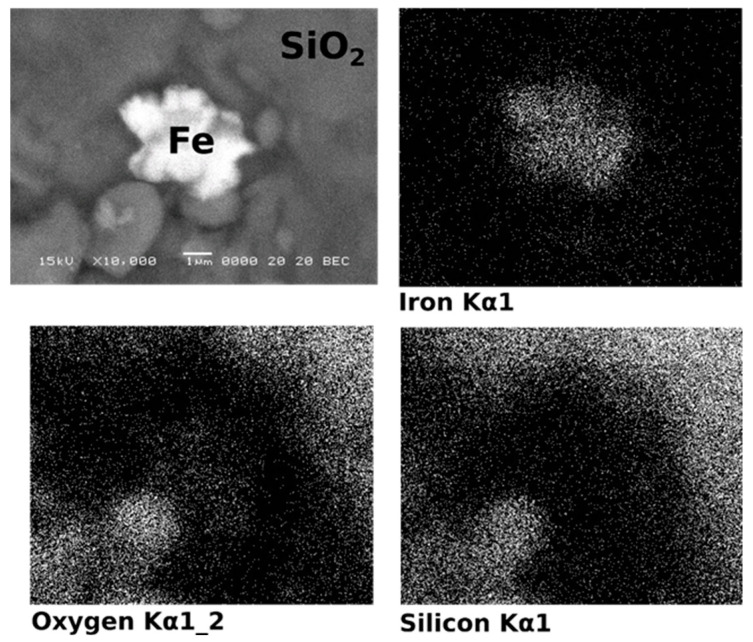
Iron particle on a larger quartz particle.

Concerning iron phases, using the elemental distribution map at the level of particle, it was found that the iron phases appear as particles attached to the larger quartz particles. This situation is illustrated in Figure 5, where the iron, oxygen and silicon elemental distribution maps are presented.

**Figure 6:**
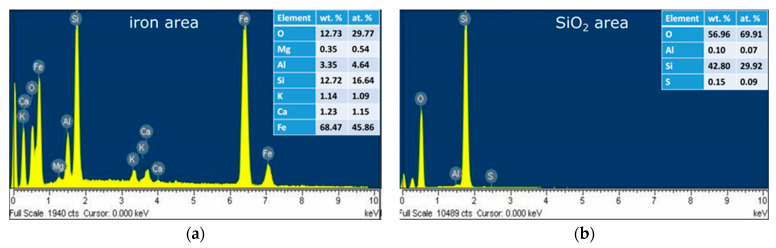
EDX spectrum and elemental quantification for the iron particle (**a**) and surrounding area (**b**).

The bright particle in the centre was identified as being composed mostly from iron. The surroundings are found to contain mostly silicon and oxygen. The EDX spectrums recorded in the two areas are presented in Figure 6.

**Figure 7:**
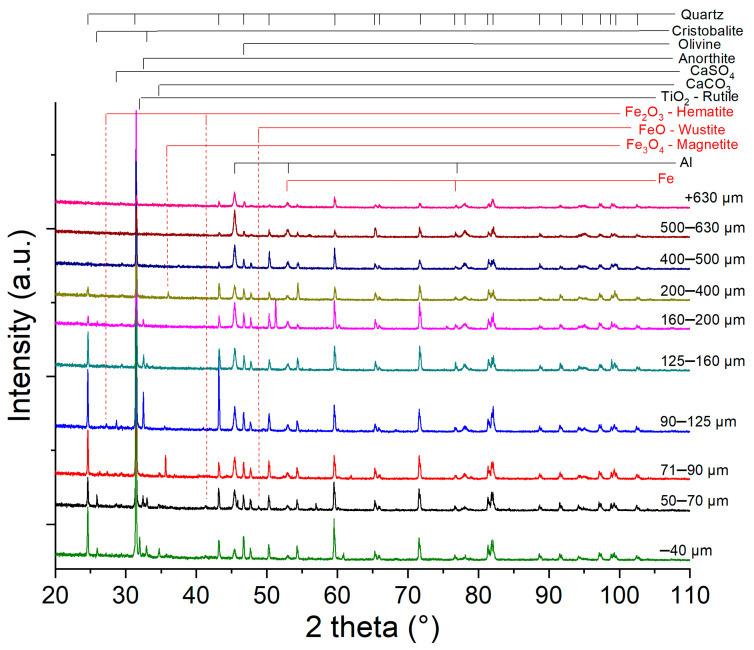
XRD diffraction patterns for different particle sizes of quartz sand.

Multiple chemical variation, recorded by EDX analysis, leads to the conclusion of crystallographic phase variation for different particle sizes. The recorded XRD patterns are presented in Figure 7.

The X-ray diffraction indicates the complex distribution of the different compounds in the sand. Al samples contain a high amount of quartz, identified as alpha phase—P3221 (PDF file nr. 00-089-8939). Iron can be found either as metallic Fe, or FeSi compound or under oxide form: Fe_2_O_3_ (hematite) and Fe_3_O_4_ (magnetite). Another element found in all samples is aluminum.

In the low angle region, additional phases are identified. As indicated by the chemical analysis, the low particle size contains titanium, identified in the XRD under the form of TiO_2_—rutile. Another compound exceeding 3% (the X-ray detection limit of a phase) is CaCO_3_. The CaCO_3_ phase is recorded in the samples particle size range 40–71 µm. For the 50 µm fraction Anorthite (Ca(Al_2_Si_2_O_8_)) is recorded. The Anorthite phase has its maximum quantity at 90 µm and decreases continuously up to 160 µm. In the sample with sizes in the 200–400 µm range, it is not detected in the X-ray diffraction patterns. Considering iron oxides, they are better detected in small size ranges. In the under 40 and 90–125 µm size ranges, samples of Fe_2_O_3_ (hematite) are recorded. The Fe_3_O_4_ (magnetite) has its maximum amount for the 71–90 µm sample. The iron phase, as observed by SEM, are very small and probably are distributed as well inside in the larger particles, but their detection is difficult. Considering the SiO_2_ phases, in all samples they appear in the form of quartz. Additional polymorphic structure is found, as stishovite, at 200–400 µm sample and cristobalite.

**Figure 8:**
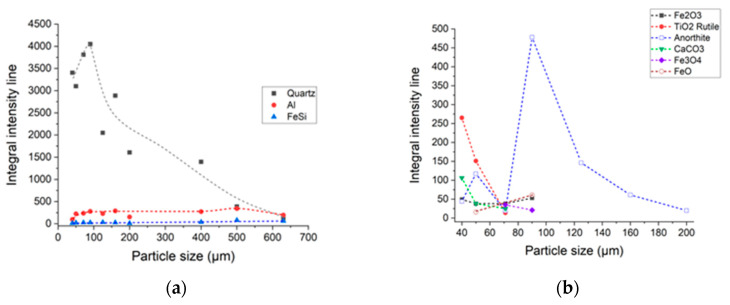
Integral peak intensity of phases presented in the sand for different particle sizes ranges: (**a**) dominant phases; (**b**) low range particle size phases.

For visualizing the relative number of phases found in the sand particles, the integral intensity of the most intense peak for each phase is presented, in Figure 8. Figure 8 confirms the hypothesis that the lower dimension particles contain important amounts of iron compounds.

**Figure 9:**
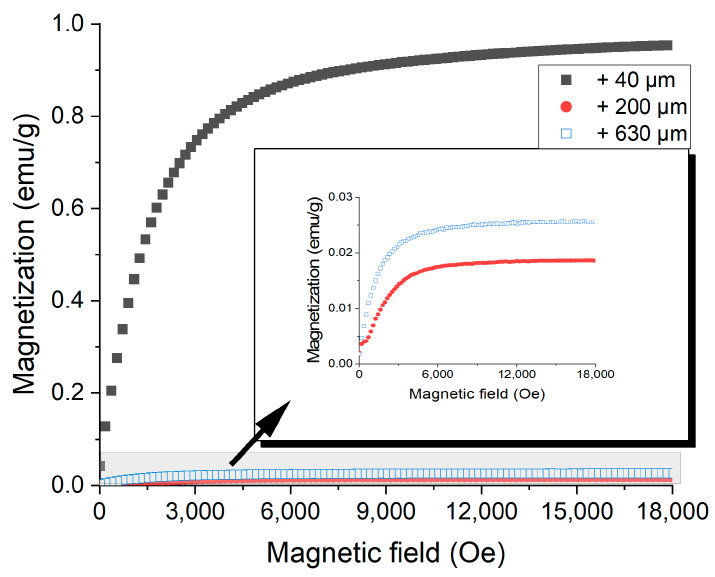
Magnetization curves and saturation induction for different quartz sand particle sizes.

For the magnetic removal of particles containing iron, the magnetization of the particles must be known. Depending on the susceptibility of different particle sizes, the efficiency of sand purification from iron phases can be adjusted to optimum. The magnetization curves recorded for several particle sizes are presented in Figure 9. Using these curves, saturation magnetization was computed.

The obtained data shows that the smallest particles (below 40 µm) possess the largest magnetization of 0.923 emu/g. The intermediate particles have the smallest magnetization of 0.018 emu/g. The spontaneous magnetization increases again for the larger particle sizes, up to 0.025 emu/g. The evolution of the magnetization follows the phase evolution recorded by XRD; lower particle sizes contain a high amount of metallic Fe or iron oxides (with higher magnetization) evenly dispersed in the sand mass. At higher particle size ranges, the particle magnetization is lower, since iron phases are diluted in larger SiO_2_ particles, as recorded by SEM/EDX. If we recall the Figure 5 image, as the iron phases seem to be mechanically blocked in the SiO_2_ particles, size reduction by further grinding can help the iron removal by magnetic separation. If the magnetization is higher, lower magnetic field values are required for separation.

**Figure 10:**
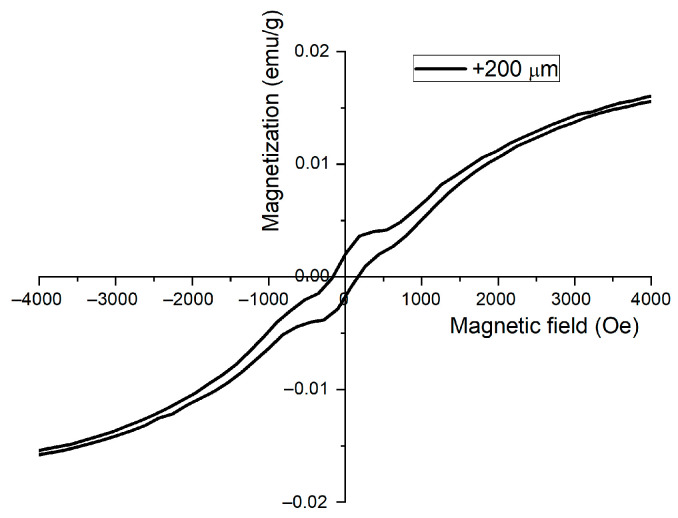
Hysteresis loop for the 200–400 µm particle size exhibiting two magnetic components.

However, for the 200–400 µm particle size, magnetization exhibits different behaviors, and a hysteresis loop was recorded, as presented in Figure 10.

**Figure 11:**
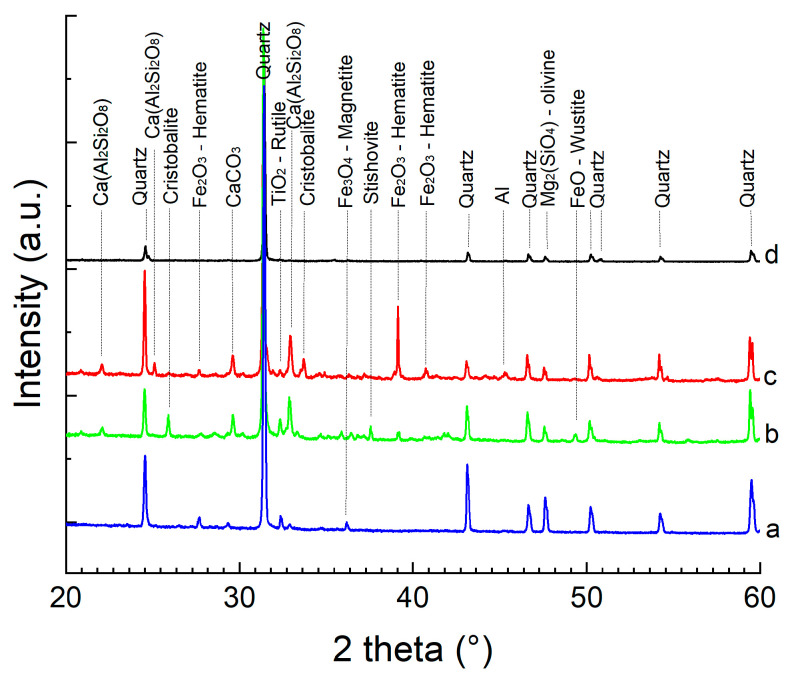
X-ray diffraction patterns recorded for the sample with 200 µm after sieving (**a**) and for the particles removed by low energy magnet (**b**), high energy magnet (**c**) and nonmagnetic fraction (**d**).

From the hysteresis loop, a bi-phasic magnetic behavior is noticed. This change indicates the presence of two magnetic components in the sand. The identification of the two magnetic components was obtained by performing a magnetic separation of the sand with two magnets presenting different energies. The magnet with low energy will attract the particles with a strong ferromagnetic behavior, while the magnet with high energy will attract the particles with weak ferromagnetic behavior. After separating the high and low magnetic component, X-ray diffraction was performed for phase identification, as depicted in Figure 11.

The sample magnetic phase removal leads to phase mixture modification. In the case of a weak energy magnet, the phase Fe_2_O_3_ (hematite) becomes more visible, alongside Fe_3_O_4_ (magnetite). Increasing the magnet energy, a very strong diffraction peak is visible for hematite; the coexistence of the two iron oxide phases being at the origin of two magnetic phases observed in the hysteresis loop.

Considering the separation part, after magnetic component removal, an overall cleaner quartz sample is obtained. Alongside the iron phases, additional phases are removed/reduced: TiO_2_, Anorthite (Ca(Al_2_Si_2_O_8_)), and CaCO_3_. The content diminution of these phases suggests their bond with the magnetic phases.

Table 2 summarizes the mineralogical analysis after two magnetic field separation of the magnetic phases contained in the quartz sand.

## 4. Conclusions

The quartz sand analyzed exhibits three major sizes: larger than 630 µm, medium sizes 200–400 µm and lower than 125 µm sizes. The iron content increases for the particle sizes smaller than 125 µm. In this region, titanium is detected; its quantity increases as the particle size decreases. The chemical distribution maps show the existence of particles with different chemical content in the low particle size region. As the particle size increases, the phases are mixed and embedded in quartz. X-ray diffraction confirms the chemical distribution analysis and indicates that iron is present in quartz as Fe_2_O_3_ (hematite), Fe_3_O_4_ (magnetite) and in lower particle size as Ilmenite (FeTiO_3_). The magnetic separation performed indicates that the iron phases are blocked mechanically in the particles and generally connected with other impurities present in quartz and are removed together. The hysteresis loop indicates the presence of two magnetic phases in the sand—Fe_2_O_3_ and Fe_3_O_4_.

## Figures and Tables

**Table 1:** Average quantitative mineralogical analysis of quartz sand particle sizes.

Particle Size Range (μm)	O (at. %)	Si (at. %)	Al (at. %)	Fe (at. %)	Ti (at. %)	Mg (at. %)	Ca (at. %)	S (at. %)	K (at. %)
40–50	71.2	23.0	2.4	0.9	0.14	0.2	1.1	0.07	0.7
50–71	71.4	23.3	2.3	0.9	0.09	0.2	0.8	0.07	0.6
71–90	72.3	22.7	2.5	0.6	0.03	0.2	0.6	0.07	0.7
90–125	72.3	23.6	2.0	0.6	0.06	0.2	0.6	0.07	0.4
125–160	73.7	21.8	2.0	0.6		0.2	0.9	0.06	0.6
160–200	73.7	21.8	2.2	0.6		0.2	0.9	0.05	0.6
200–400	73.4	22.7	1.8	0.6		0.2	0.6	0.07	0.5
400–630	72.5	23.5	1.6	1.00		0.2	0.7	0.08	0.3

**Table 2:** Average mineralogical analysis of 200–400 μm particle size range by magnetic separation with low and high energy magnet.

Particle Size Range (μm)	O (at. %)	Si (at. %)	Al (at. %)	Fe (at. %)	Ti (at. %)	Mg (at. %)	Ca (at. %)	S (at. %)	K (at. %)	Na (at. %)	Mn (at. %)
Unseparated particles	70.9	26.1	1.3	0.4	-	0.1	0.5	0.04	0.6	0.2	-
Low energy magnet	69.3	19.9	3.8	2.8	0.3	1.3	1.3	0.05	0.8	0.8	-
High energy magnet	67.9	19.2	4.4	3.8	0.4	1.1	1.8	0.06	0.6	0.7	0.2
Nonmagnetic particles	70.6	26.8	1.1	0.3	-	0.1	0.4	0.03	0.5	0.2	-

## Data Availability

The authors confirm that the data supporting the findings of this study are available within the article.
